# RAIphy: Phylogenetic classification of metagenomics samples using iterative refinement of relative abundance index profiles

**DOI:** 10.1186/1471-2105-12-41

**Published:** 2011-01-31

**Authors:** Ozkan U Nalbantoglu, Samuel F Way, Steven H Hinrichs, Khalid Sayood

**Affiliations:** 1 Department of Electrical Engineering, 209N WSEC University of Nebraska-Lincoln, Lincoln, NE 68588-0511 USA; 2 Department of Pathology and Microbiology, University of Nebraska Medical Center, Omaha, NE 68198 USA

## Abstract

**Background:**

Computational analysis of metagenomes requires the taxonomical assignment of the genome contigs assembled from DNA reads of environmental samples. Because of the diverse nature of microbiomes, the length of the assemblies obtained can vary between a few hundred bp to a few hundred Kbp. Current taxonomic classification algorithms provide accurate classification for long contigs or for short fragments from organisms that have close relatives with annotated genomes. These are significant limitations for metagenome analysis because of the complexity of microbiomes and the paucity of existing annotated genomes.

**Results:**

We propose a robust taxonomic classification method, RAIphy, that uses a novel sequence similarity metric with iterative refinement of taxonomic models and functions effectively without these limitations. We have tested RAIphy with synthetic metagenomics data ranging between 100 bp to 50 Kbp. Within a sequence read range of 100 bp-1000 bp, the sensitivity of RAIphy ranges between 38%-81% outperforming the currently popular composition-based methods for reads in this range. Comparison with computationally more intensive sequence similarity methods shows that RAIphy performs competitively while being significantly faster. The sensitivity-specificity characteristics for relatively longer contigs were compared with the PhyloPythia and TACOA algorithms. RAIphy performs better than these algorithms at varying clade-levels. For an acid mine drainage (AMD) metagenome, RAIphy was able to taxonomically bin the sequence read set more accurately than the currently available methods, Phymm and MEGAN, and more accurately in two out of three tests than the much more computationally intensive method, PhymmBL.

**Conclusions:**

With the introduction of the relative abundance index metric and an iterative classification method, we propose a taxonomic classification algorithm that performs competitively for a large range of DNA contig lengths assembled from metagenome data. Because of its speed, simplicity, and accuracy RAIphy can be successfully used in the binning process for a broad range of metagenomic data obtained from environmental samples.

## Background

A principal goal of metagenomics [1] is to sample microbiomes and recover genetic material without isolating single organisms, thereby mitigating the problem of limiting genomic analysis to a small percentage of existing culturable species. Eventually, this will help extend the tree of life [2], enrich sequence libraries, and expand analysis from genomic to metagenomic (e.g., samples from various habitats could be used to study interactions within communities, determining the ecological and metabolic roles of microbes in a community, their lifestyles, and evolution).

Recent advances in genome sequencing technology have made metagenomics more feasible. Second generation sequencing technology, such as Roche 454, ABI SOLiD, and ILLUMINA [3,4], provide a greater amount of sequenced data at lower cost. Sequencing metagenomes sampled from their natural habitat is already physically achievable. However, there are important computational challenges. Complete assemblies of genomes in environmental samples are rarely achievable for several reasons.

The first obstacle is the population characteristics of the microbiome communities. While dominant species are represented in the samples by a greater number of clones, proportional to the abundant number of individuals, less abundant species are sparsely represented or in some cases, not captured due to incomplete sampling [5], resulting in their low representation, or absence, in the metagenome. The assembled contigs from metagenome reads are generally shorter than the contigs obtained from the sequencing of single genomes. The average contig length is frequently around a few kilobase pairs (kbp). For high diversity metagenomes, much of a metagenome cannot be assembled, leaving the majority of the reads as singletons. For example, in a diverse community sample obtained from Minnesota Soil, less than 1% of the reads could be assembled [6].

The second obstacle arises from the fact that the new sequencing technologies generate shorter reads. In currently popular sequencing technologies, a read corresponds to DNA fragments with lengths between 100-800 bp creating greater dificulties in assembling contigs as well as shorter singletons. All in all, these technologies yield contigs ranging from a few hundreds of base pairs to a few tens of kbp at the end of the metagenome assembly process.

Analyzing the composition of such a large amount of fragmented data remains a computational challenge. In order to identify which operational taxonomic units (OTUs) exist in the mixture, **phylotyping **methods are often employed that involve searching for 16 S rRNA marker genes [7] or a set of marker genes [8] in a metagenome to locate the OTUs in the existing phylogeny trees. AMPHORA [9] and MLTreeMap [10] use marker gene analyses to infer the phylogenetic information of a given environmental sample. While those programs supply information about the biodiversity of a sample, they only associate those genome fragments that carry a marker gene with possible OTUs. That means the great majority of sequencing reads remain unassociated with any taxa. However, along with the composition of the metagenome, classifying each sequence fragment into taxonomic units is also a challenge that needs to be met in metagenomics. This is required to link genes discovered to the members of a taxon or to enable divide-and-conquer approaches as a solution to the metagenome assembly problem.

In this article, we refer to the process of classifying the metagenome fragments into OTUs as **binning**. We can categorize most binning methods as **similarity-based methods **and **composition-based methods**. In similarity-based methods, the DNA fragments to be classified are compared with existing sequences by performing string similarity searches against a molecular sequence database (e.g., BLAST). The MEGAN system [11] is an example of similarity-based binning. It attempts to predict the source of the sequences by assigning them to hypothetical common ancestors using multiple high scores gathered from a similarity search system, such as BLAST [12]. CARMA [13] is an algorithm that assigns the sequences to taxonomical origins by trying to match them to known protein families contained in Pfam domains. Although these methods are frequently used for phylotyping, they can be employed for binning since they utilize comprehensive protein/nucleotide domains and attempt to classify any given genome fragment. While computationally expensive, these algorithms have been shown to be accurate even for short sequences in the current pyrosequencing read length range (80-400 bp). However, the accuracy drops dramatically when phylogenetically close sequences are missing from the search databases. Running CARMA on a comprehensive dataset gathered from a large spectrum of known genomes resulted in inaccurate classifications [13]. (6% sensitivity when using 100 bp sequences for identification at the genus level). Moreover, in a recent study [14], only 12% of the data obtained from microbial communities in coral atolls got significant BLAST hits. Thus, even if similarity-based methods were much more accurate, they would still be unable to identify sequences from a large proportion of the microbial population.

Composition-based binning methods approach the problem using conserved compositional features of genomes, such as short oligonucleotide usage patterns. It has been observed that relative frequencies of k-mers in DNA sequences are taxon specific and carry phylogenetic signals. Moreover, they are fairly conserved for relatively short fragments. Thus to some extent, it is possible to predict the origin of a random DNA fragment given the relative oligonucleotide frequencies of the possible sources. A number of unsupervised methods for clustering fragments originating from similar taxonomic units have been proposed. TETRA [15] uses relative proportions of tetranucleotides with respect to the database samples in DNA contigs and calculates the correlations of pairs as a measure of similarity. Self-organizing maps (SOM [16,17], GSOM [18], S-GSOM [19]) are used for clustering profiles of tetranucleotide frequencies.

When the binning task includes assigning taxonomical labels using available genomic information, supervised methods are employed; and the available genomic sequences of known organisms are used for training source models. A naive-Bayesian Classifier-based method proposed by Sandberg et al. [20] and a Markov chain method by Dalevi et al. [21] are early examples of this approach. The algorithm, PhyloPythia [22], consists of various support vector machine (SVM) Classifiers and uses relative frequency profiles of short oligonucleotides as feature vectors. Satisfactory sensitivity and specificity results are reported for the sequence lengths >1-3 kbp. However, a sharp cutoff in the accuracy is observed for fragments less than 1 kbp in length. Another recent taxonomic classification method, TACOA [23], proposes a k-nearest-neighbor-classification-based algorithm. In this method, genomic sequences are represented by the abundance profiles of oligonucleotides. TACOA correctly classifies fragments larger than 800 bp with an average sensitivity between 76% at rank superkingdom and 39% at rank genus, and its performance is comparable to PhyloPythia in that range.

There are several ways of measuring the distance or similarity of sequence composition, in particular the distances between relative oligonucleotide frequencies or oligonucleotide abundance feature vectors. However, measures such as Euclidean distances (PhyloPythia), inner products (TACOA), and Pearson Correlations (TETRA) have some inherent limitations in this context. Since all 4*^k ^*oligonucletotide components calculated over k-mer frequencies are involved in similarity measurements, good estimates of all components are necessary; and overfitting dramatically drops the detection accuracy. In order to prevent overfitting, more data points are needed to accurately estimate frequencies of occurrence (i.e., longer fragments); and the number of parameters to be estimated should preferably be kept small (i.e., shorter oligonucleotides). Not surprisingly, all of these methods work best with relatively short oligomers (TETRA, TACOA: 4-mers, and PhyloPythia: 5-mers) with sequences ≥1-3 kbp. However, longer-range correlations exist in DNA sequences, which we would like to exploit for fragment identification even with short sequence reads. It has been observed that methods that employ probabilistic frameworks, such as the naive Bayesian Classifier, which estimates the probability of observing DNA sequences based on relative oligonucleotide frequencies [20], or Markov models [21,24], can achieve better accuracy for longer oligonucleotides, even for sequences shorter than 3 kbp.

To date, the only composition-based algorithm reported to be successful with short reads is Phymm [24]. It was developed for the classification of short read lengths of metagenomics data. It is based on a Bayesian decision machine that detects the taxonomic source of a read with its maximum a posteriori probability calculated over variable-order Markov models. Phymm provides significantly better accuracy than CARMA and PhyloPythia. PhymmBL [24], which combines the Phymm-based approach with a BLAST-based similarity approach, provides superior performance to Phymm, albeit at a significant computational cost.

Metagenomic fragment classification remains an important computational challenge that requires the taxonomic assignment of genomic sequences of various lengths, which in turn requires reliable sensitivity and specificity values for a large spectrum of fragment lengths. We propose a composition-based semisupervised binning algorithm, RAIphy. This method characterizes, or models, a genome/taxonomic unit with a set of parameters called the Relative Abundance Index (RAI). According to this model, an index value is calculated for each k-mer, based on the over- and underabundance statistics gathered from the taxon. Unlike the work of Brendel et al. [25] used in the work of Qi et al. [26] and Wan et al [27], the over- and underabundance statistics are obtained with reference to a sequence of Markov models. A given random genome fragment is given a membership score with respect to a taxon by adding up the index values in the RAI model for the taxon for each observed k-mer in the fragment. The fragment is assigned to the taxon that results in the highest score. An iterative process consisting of classifying the fragments from a mixture using the current RAI models then updating the RAI models based on the resulting clusters is used to improve the classification accuracy. As the initial RAI seeds, RAIphy uses models estimated from genomes currently available in the RefSeq database, and thus RAIphy can be categorized as a semi-supervised method.

RAIphy has been implemented as a simple, compact standalone desktop application, which is fast compared to similarity-search-based applications. While achieving competitive binning accuracies for the DNA sequencing read length range (100-1000 bp), the method also performs accurately for longer environmental contigs.

## Methods

There are three components of the RAIphy algorithm: the Relative Abundance Index and the corresponding profile or model for genomic fragments and taxonomic units; the classification metric; and the iterative algorithm used to refine the models used for classification. We describe each in turn.

### Relative Abundance Index

The **Relative Abundance Index **is a measure of the relative abundance of oligonucleotides in genomic fragments. Based on the available genomic sequences for a taxonomic unit, a score is assigned to each possible k-mer. These scores are higher for overrepresented k-mers and lower for underrepresented k-mers. The vector of k-mer scores comprises the RAI profile for a taxonomic unit.

The under and the overrepresentation of each k-mer is measured using the log-odd ratios between the observed and expected frequencies of each k-mer. Therefore, for an overrepresented k-mer, the observed frequency is higher than the expected frequency; and the RAI score is positive. It is negative for an underrepresented k-mer. Using relative frequency counts as estimations of k-mer probabilities in a genome sequence, we use Markov assumptions for the calculation of expected k-mer probabilities.

Consider a *k*-mer, *x_1_, x_2_, . . . , x_k_*, with probability p(*x_1_, x_2_, . . . . x_k_*). We can write this probability as:

(1) $$p(x_{1},x_{2},\ldots ,x_{k})=p(x_{k}|x_{1},x_{2},\ldots ,x_{k-1})p(x_{1},x_{2},\ldots ,x_{k-1}).$$

We can rewrite the first factor on the right hand side of Equation (1) under different independence assumptions as follows. Assuming that the bases occur independently of each other, the conditional probability can be replaced by the marginal probability

(2) $$p(x_{k}|x_{1},x_{2},\ldots ,x_{k-1})=p(x_{k}).$$

To test this assumption, we can compute a log odds ratio to form the relative abundance index of order 0 *rai*_0_:

(3) $$rai_{0}(x_{1},x_{2},\ldots ,x_{k})=\log_{2}\frac{p(x_{1},x_{2},\ldots ,x_{k})}{p(x_{k})p(x_{1},x_{2},\ldots ,x_{k-1})}.$$

If we assume that the bases follow a first order Markov model,

(4) $$p(x_{k}|x_{1},x_{2},\ldots ,x_{k-1})=p(x_{k}|x_{k-1})$$

(5) $$=\frac{p(x_{k-1},x_{k})}{p(x_{k-1})}.$$

The corresponding relative abundance index *rai*_1 _is then given by:

(6) $$rai_{1}(x_{1},x_{2},\ldots ,x_{k})=\log_{2}\frac{p(x_{1},x_{2},\ldots ,x_{k})p(x_{k-1})}{p(x_{k-1},x_{k})p(x_{1},x_{2},\ldots ,x_{k-1})}.$$

Continuing in this fashion, we obtain:

(7) $$rai_{i}(x_{1},x_{2},\ldots ,x_{k})=\log_{2}\frac{p(x_{1},x_{2},\ldots x_{k})p(x_{k-1}\ldots x_{k-i})}{p(x_{k},x_{k-1},\ldots x_{k-i})p(x_{1},x_{2},\ldots x_{k-1})}.$$

We combine the relative abundance indices of all orders by adding them to give the total superposed profile

(8) $$rai(x_{1},x_{2},\ldots ,x_{k})=\sum_{i=0}^{k-2}rai_{i}(x_{1},x_{2},\ldots ,x_{k}).$$

Given a particular *k*-mer, *x_1_, . . . , x_k_*, {*rai*(*x*_1_, *x*_2_, . . . , *x_k_*)} is a positive value if the probability of observing *x*_1_, . . . , *x_k _*is higher than expected for a particular taxonomic unit, and a negative value if the probability of observing *x*_1_, . . . , *x_k _*is lower than expected for a particular taxonomic unit.

The probabilities used for obtaining the RAI values for k-mers are estimated using the relative frequency of occurrence of that k-mer in the taxonomic unit. Thus, for each taxonomic unit for which we have genomic sequences, we compute a corresponding RAI profile. This profile serves as a model for that taxonomic unit.

### Classification Metric

To assign a genomic fragment, *F*, from an unknown source to a taxonomic unit, we first compute the relative frequencies of occurrence for each k-mer from the fragment. For each candidate taxonomic unit, we then obtain a membership score by computing the weighted sum of the components of the RAI profile of the taxonomic unit where the weighting is the corresponding k-mer frequency of occurrence for the fragment *F*.

Given a RAI model belonging to the taxon, *G_i_*, and an unknown genome fragment, *F*, the membership score, *E_F _*[*rai^Gi ^*], is given as:

(9) $$E_{F}[rai^{G_{j}}]=\sum_{\text{x}}f_{F}(x_{1},x_{2},\ldots ,x_{k})rai^{G_{j}}(x_{1},x_{2},\ldots ,x_{k}),$$

where *f_F _*(*x*_1_, *x*_2_,..., *x_k_*) is the frequency of a k-mer in the fragment, *F*, and *rai^Gj ^*is calculated using the relative frequency counts of the k-mers observed in the taxon, *j*. Consider what happens when the statistics of the k-mers of the fragment match the statistics of a taxonomic unit. For a k-mer that occurs often, the frequency of occurrence will be a high and the RAI value of the k-mer for the taxonomic unit will be positive. The more often the k-mer occurs, the larger will be the values of both the RAI and the frequency of occurrence. For k-mers that occur less often than expected, the frequency of occurrence will be low; and the RAI value of the k-mer for the taxonomic unit will be negative. Thus in the sum, the positive RAI values will be weighted by the larger frequencies of occurrence; and the negative values will be weighted with the lower frequencies of occurrence. The opposite will happen when the statistics of the fragments are completely mismatched with the statistics of a taxonomic unit. Therefore, the membership score for the matching taxonomic unit will be higher than the membership score for the mismatched taxonomic unit. We have empirically observed that genome fragments attain higher membership scores when they share the same taxa with an RAI profile (Additional File 1). This can also be shown mathematically assuming stationarity (Additional File 2).

Given the taxa, *J *= {1, 2,...,*n*}, with RAI profile. $\}rai^{G_{1}},rai^{G_{2}},\ldots ,rai^{G_{j}},\ldots ,rai^{G_{n}}\}$, an unknown genome fragment, *F*, is classified to the taxon, $\overset{⌢}{j}$, by

(10) $$\overset{⌢}{j}=\arg\underset{j}{\max}E_{F}[rai^{G_{j}}].$$

We have observed that RAI performs better than the similarity measures defined by Sandberg et al. [20] and Dalevi et al. [21] with the same experiment setup used in those studies (Additional File 3). Therefore, we have adopted RAI as the compositional detection approach to be used in our metagenomic phylogeny classification.

### Iterative Refinement of Genome Models

Metagenomics binning programs are designed for classifying genome fragments of previously unknown species using phylogenetically close genomes. Since the conserved compositional features, or genome signatures, of the unknown species in the mixture are not available, the presumption is that the classification algorithm will assign the fragment to the same or a close clade-level for which a model (in this case an RAI profile) is available. While this can be done with some success, there remains significant room for improving the classification accuracy by adaptively updating the models used for detection. The heuristics presented here rely on the fact that we actually possess genomic fragments from the unknown genome in the mixture. Therefore, we use a multistep process in which the first step uses classification, as described above, using the RAI profiles of known species. Once this first classification has been performed, the resulting clusters of fragments can be used to obtain the RAI profiles of the unknown species. Obtaining the genome signatures of these clustered fragments (and subsequently training models over them) results in models that better describe the composition of the unknown genome leading to more accurate classification. Experiments supporting these claims are presented in the Results section.

The refinement procedure consisted of the repetition of two phases. In the first phase, RAI profiles were estimated from genomes of known organisms. Each metagenome fragment was classified by assigning it to the genomes returning the maximum RAI score. In the second phase, the oligonucleotide frequencies and, subsequently, the RAI profiles for each class were recalculated using the collection of fragments assigned to the corresponding class. These two phases were iteratively repeated until a stopping criterion was met. With each refinement, the metagenome fragments were represented with improved RAI profiles. Thus, the average membership scores were expected to increase. When the change in the increase of average membership scores with a refinement became small, we stopped the refinement procedure. Here, the stopping criterion was met if the improvement in the score was less than 1% of the membership score achieved in the previous iteration. The algorithm is quite robust to the stopping threshold; reducing the threshold by several orders of magnitude has no effect on the binning performance. This procedure can be thought of as an expectation maximization algorithm with hard decision of classes [28]. From this point of view, it is similar to a seeded K-means clustering algorithm, with training initial conditions using previously known data [29]. Instead of minimizing the mean Euclidean distance, our objective was to maximize the mean average membership score. The algorithm can be summarized as follows:

Classification with iterative refinement:

*N Metagenome fragments*: *F_j_j *∈ {1, 2, .., *N*}

*M RAI profiles: rai^Gi ^i *∈ {1, 2, .., *M*}

*M *taxonomic classes: *G_i_i *∈ {1, 2, .., *M*}

1. CLASSIFY all*F_j _*using all*rai^Gi^*

2. UPDATE all*rai^Gi ^*using*F_j _*∈ *G_i_*

3. BREAK IF$|\frac{AVERAGE\_Membership\_SCORE\_CURRENT}{AVERAGE\_Membership\_SCORE\_PREVIOUS}-1|<0.01$

4. GOTO 1

**Figure 1:**
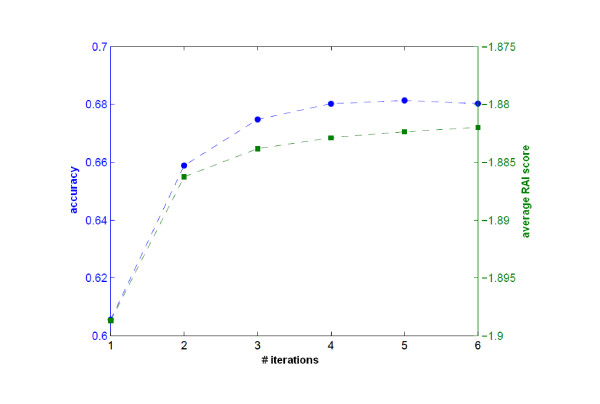
**Iterative refinement**. The performance increase with iterative refinement is illustrated using the same dataset and experiment setup with [24] for the fragments of length 400 bp. *Left y-axis and blue curve: *The increase in the percent of correct assignments with iterative refinement. *Right y-axis and green curve: *The increase and saturation in the average relative abundance index scores.

We tested the performance of this algorithm using the same data and experimental design as in [24] (i.e., the same genomes were used for training RAIs, and the same fragments were used for testing). The test fragments in this dataset were short fragments in the range of 100-1000 bp. Observing the performance of iterative refinement on short fragments was important because the ratio of false positives is greater for short fragment lengths, as is the noise introduced by them. Therefore, the task of improving the models in this band was harder. We observed improvement in classification accuracy for all fragment lengths we tested in a small number of iterations (3-6). The increase in accuracy for the fragment length of 400 bp is shown in Figure 1.

### Test Data

In order to be able to conduct controlled experiments, we created synthetic metagenome data using the available genomes in the US National Center for Biotechnology Information (NCBI) RefSeq database [30] as of March 2010. We built our database storing RAI profiles for all 1,146 available genomes. Different chromosomes and plasmids belonging to the same organism were concatenated and treated as a single sequence. These served as the initial seeds in a run of RAIphy. For phylogenetic binning and labeling, we collected the taxonomic information from the NCBI taxonomy database. The data collected was comprised of 609 species, 318 genera, 158 families, 88 orders, 41 classes, and 26 phyla. To test the performance of our program, leave-one-out, cross-validation tests were performed as follows: for every taxonomic unit comprised of at least two subtaxa (e.g., a genus having more than one different species), a test genome was selected; and 3000 test fragments were drawn randomly from each one of those genomes. The RAI profiles were trained over the remaining taxa. The test genome was not used for obtaining the RAI profile. This was done for every genome that was not a single representative of a clade. We repeated each experiment 100 times to assess the first and second order accuracy statistics.

### Program Parameters

**Figure 2:**
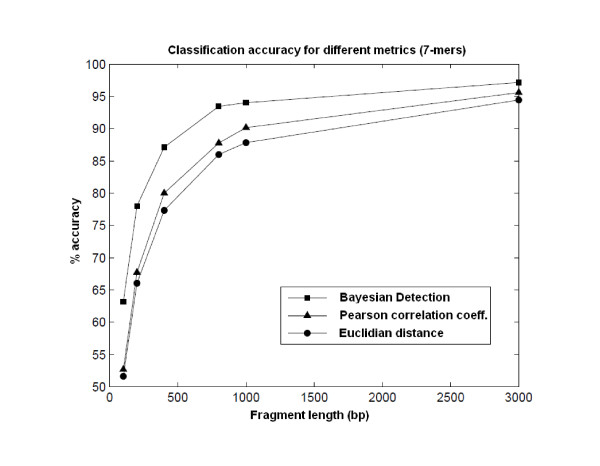
**Classification accuracy for different distance metrics**. The accuracy performance of different distance/similarity metrics for 28 taxa with varying fragment lengths is shown. The frequencies of 7-mers were used. Using the metric defined by Sandberg et al. appears to be more accurate for all fragment lengths than employing Euclidean distance and Pearson correlation coefficients.

Since RAIphy was designed as an iterative algorithm, which retrains its models depending on the change in the average membership score, the parameters were kept constant for the whole spectrum of fragment lengths. The oligonucleotide length was fixed at seven. Although it has been shown that longer correlations exist in DNA and that it is possible to exploit longer oligonucleotides for sufficient sequence lengths [31], we observed that the classification accuracy saturates after an oligomer length of seven (Additional File 3). The binning accuracy increases significantly with the increase in k-mer size to a size of seven (Figure 2, Additional File 3). However, increasing the size of the k-mers beyond seven results in negligible accuracy improvement while significantly increasing the computational burden. An RAI profile was updated only if the total length of the fragments assigned to the corresponding class exceeded 25 kbp.

## Results and Discussion

### Absolute Metrics vs. Probability Metrics

In order to show the improvement in accuracy obtained by replacing the distance/similarity metrics from absolute metrics, such as Euclidean norms, or correlation coefficients to probabilistic measures, such as Bayesian posterior probabilities, we ran the same experiments with different metrics using the dataset in [20]. The example shows the percent of true positive ratios for short fragments, and the metric defined in Equation [20] can be observed to be more accurate for 7-mer frequencies. This was the motivation for using a probabilistic method for oligonucleotide usage measurement. The results are shown in Figure 2. The improvement of Bayesian detection over the use of Euclidean distance and the Pearson correlation coefficient is evident for all fragment lengths.

### Experiments in Support of the Refinement Process

**Figure 3:**
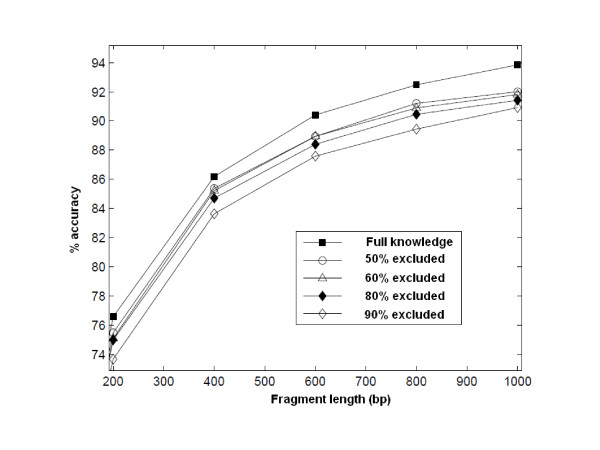
**Classification accuracy for partial genome knowledge**. Classification accuracy performance with varying available coverage of training genomes. RAI profiles are built using the entire genome and fragments of genomes covering 50%, 40%, 20%, and 10% of the genome. The decrease in the classification performance due to incomplete training data coverage was not significant, and classification capability was conserved.

There are two observations that support the thesis that a refinement process will improve the overall detection performance. First, the genome signatures estimated using the detected portion of a genome should be a good approximation of the signature of the unknown genome. That is to say, we should be able to perform sufficient classification with the models trained from incomplete genomes and even with a collection covering a small percentage of the genome. Although the genome signatures are known to be pervasive, we investigated whether the pervasiveness was sufficient to allow a reasonable estimate of the signature to be extracted from a small fraction of the genome. We repeated the fragment classification experiments in [20] using models trained over various coverage percentages of genomes starting from the entire genome down to only 10% of the genome. Employing the RAI in the manner described above, as shown in Figure 3, we observed that there is only a decrease in accuracy of 2-4% in the worst case. This result supports the premise that even with a small collection of fragments in a taxonomic bin after the classification we could train a practically useful model for the unknown organism.

The first experiment demonstrated that it was possible to train a model with a small fraction of the genome that could be obtained through classification of the samples of the microbiome. However, these results assume that the genomic fragments available truly belong to the organism being detected. Taxonomic classification algorithms return significant amounts of false positives. These false positives could conceivably make the algorithm diverge and actually reduce classification accuracy. We conducted a number of experiments to make sure that this would not happen with RAIphy for the metagenomic classification experiments. An example of the results of such an experiment is shown in Figure 1. We had no experiments in which the algorithm diverged.

### Classification Performance for Short Fragments

**Figure 4:**
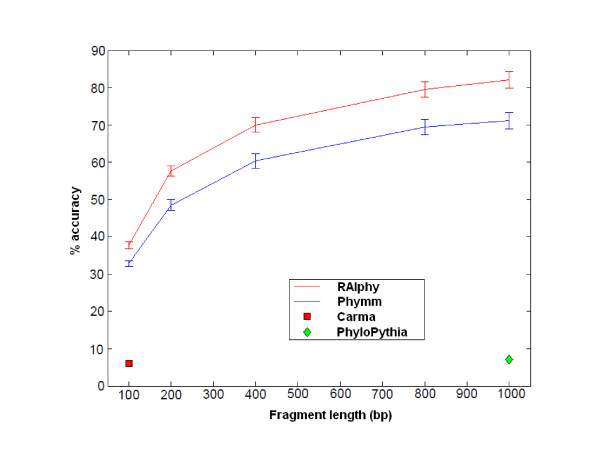
**Read length range accuracy**. Accuracy of RAIphy with short fragment lengths and genus-level prediction, compared with Phymm in the same spectrum. PhyloPythia operates accurately for >1000 bp fragments. Here, its poor performance for short-read range can be observed for 1 Kbp accuracy. Also, Carma searching Pfam domains and protein families for short reads, such as 100 bp fragments, appeared to be performing poorly in accordance with the results reported in [13].

**Figure 5:**
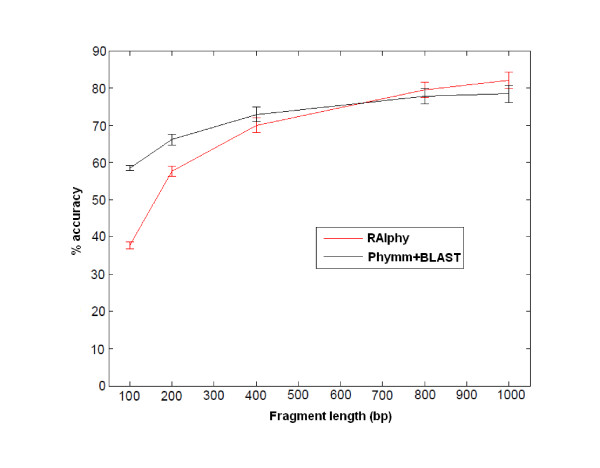
**Read length range accuracy for RAIphy vs Phymm combined with BLAST (PhymmBL)**. Accuracy of RAIphy with short fragment lengths and genus-level prediction, compared with PhymmBL in the same spectrum. For short read length (100 bp-400 bp) fragments, the combination of Phymm and BLAST outperforms RAIphy. However, RAIphy attains higher accuracy for longer fragments.

The first set of experiments included testing the accuracy of RAIphy for short fragments in the range of 100-1,000 bp. The experiments were divided into ranges or bands of fragment length, because existing programs operating in different bands have different accuracy scores and properties. For example, TACOA and PhyloPythia perform poorly for short fragments as mentioned above. On the other hand, similarity-based programs, such as Carma, also perform poorly when the genome of origin is not available. Currently, the only composition-based method that can accurately classify previously unobserved metagenome samples in this range is Phymm. In Figure 4, the accuracy (i.e., the percent true positive rate) performance with changing fragment lengths is illustrated. It can be seen that the RAIphy classification performance compares favorably to Phymm for all fragment lengths. In Figure 5, RAIphy is compared with PhymmBL, which combines Phymm and BLAST. PhymmBL outperforms RAIphy for shorter fragment lengths at a cost of significantly increased computation time.

### Binning Fragments in the Absence of Close Relatives

Even with our contemporary knowledge of microbiology, a great majority of the tree of life is unknown. Therefore, it would not be unexpected to have genome fragments of an unknown clade in a metagenome sample. In this case, a metagenome binning method is desired to assign the fragments of undiscovered genomes to sister taxa in the same clade-level. To simulate this situation and observe how RAIphy performs in such cases, we tested it with incomplete training data. We repeated the previous experiments with leave-one-out, cross-validation; however, this time, all representatives of the taxonomic group that the test samples belong to were removed from the training data and an assignment to a sister taxon (e.g., a genus from the same family with the unknown genus) was accepted as a correct classification. We performed the tests for the unknown taxa of different clade-levels from family to class levels.

**Figure 6:**
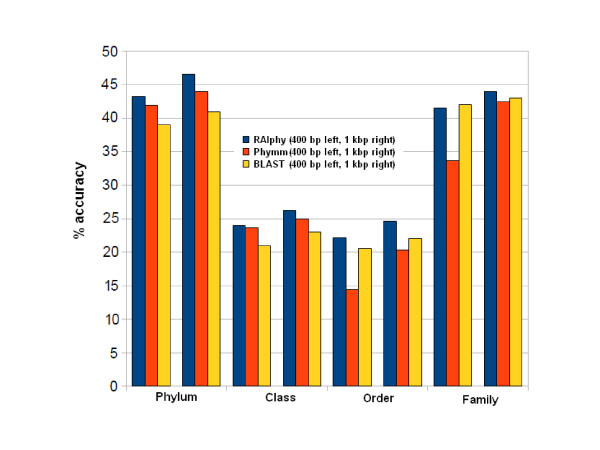
**Binning performance in the Absence of Close Relatives**. Comparison of RAIphy, BLAST and Phymm with incomplete training set for varying clade-levels is shown for 400 bp, 1 Kbp genomic fragments. The accuracy remains under 50% for all methods. RAIphy performs slightly better than Phymm and BLAST for this range.

The correct classification rate decreased substantially with missing data. RAIphy performed at under 50% accuracy for all clade-levels for fragment lengths in the range of 100 bp-1 Kbp. In Figure 6, the binning performance for RAIphy, Phymm, and BLAST searches is illustrated for a read length of 400 bp and 1 Kbp. While this performance is still superior to other composition-based methods, similarity searches performed using BLAST performed better for short read lengths of 100 bp and 200 bp (Additional File 4).

### Classification Performance for Longer Metagenome Fragments

The classification performance for genomic fragments of 800 bp-50 Kbp was also studied. This range is significant because it represents lengths of assembled contigs, while the shorter fragments correspond to single sequencing reads. In taxonomic classification, generation of a smaller number of highly reliable predictions is preferred over predicting the majority of fragments with less reliable labels [23]. When this is the case, genomic fragments with reliable scores can be classified and suspicious fragments left as "unknown." Adopting the accuracy measurement definitions defined by Baldi et al. [32], this kind of regularization yields higher average specificity and lower average sensitivity. The sensitivity for the class *i *is defined as:

(11) $$Sn_{i}=\frac{TP_{i}}{TP_{i}+FN_{i}+U_{i}},$$

where *TP_i _*is the number of samples correctly classified to the class *i *(*true positives*), *FN_i _*is the number of samples assigned to another class even though they belong to class *i *(*false negatives*), and *U_i _*is the unclassified number of samples belonging to class *i*. The specificity for the class *i *is defined as:

(12) $$Sp_{i}=\frac{TP_{i}}{TP_{i}+FP_{i}}$$

where *FP_i _*is the number of samples assigned to the class *i *while belonging to another class.

Determining an operating point in the sensitivity-specificity trade-off was achieved by using different approaches for different methods. In TACOA, the kernel parameters governed the thresholds for classifying samples. In Diaz et al. [23], grid searches were employed to decide the optimal accuracy values and for setting the parameters. PhyloPythia uses a post-processing one-versus-all SVM Classifier to detect the reliable samples and leave the rest "unknown." RAIphy classifies all metagenomic fragments to a taxonomic bin by default. However, RAIphy also allows setting thresholds and operating at different points of the sensitivity-specificity curves. We assigned detection-quality scores to fragments to measure the likelihood of fitting. The quality scores were calculated as the difference between the best average RAI score and the next best score:

(13) $$q\left(F\right)=E_{F}[rai^{G_{i}}]-E_{F}[rai^{G_{k}}]$$

where *i *is the class returning the best RAI score (*E_F _*), and k is the class returning the second highest RAI score. If a fragment fits equally well to more than one model, the quality score turns out to be 0; and if a fragment reflects the characteristics of one class much better than any other class, it receives a high quality score.

Setting percentage thresholds (*p*) to assign the top *p*% scored fragments of each class and drop the labels of the remaining (100-*p*) % to "unknown" increased the specificity while reducing the sensitivity. Geometrically speaking, the fragments remaining in an iso-quality hyperboloid were assigned; and the others outside the hyperboloid were determined to be unclassified. Therefore, this thresholding is a tightening of the decision boundary from a hyperplane to a hyperboloid in the feature space.

**Figure 7:**
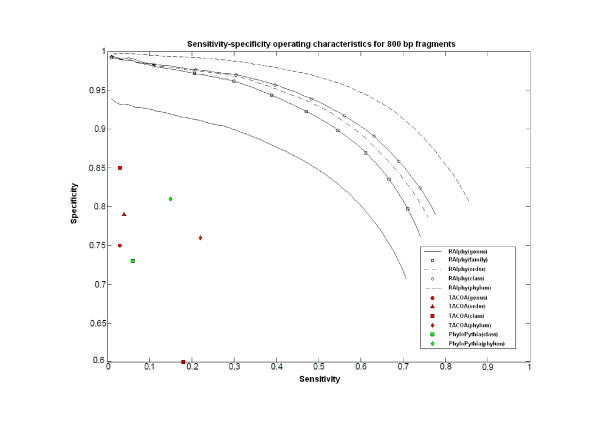
**Sensitivity-specificity operating characteristics (800 bp)**. Sentitivity-specificity operating characteristics curves for RAIphy determined with 800 bp fragments using the dataset obtained from the RefSeq database. The accuracy values for TACOA and PhyloPythia are also illustrated for the same test data.

**Figure 8:**
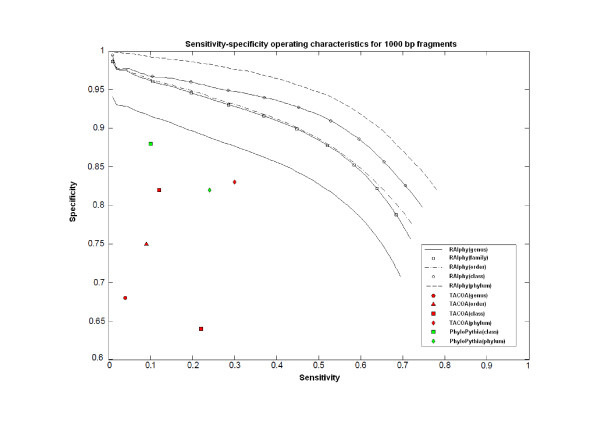
**Sensitivity-specificity operating characteristics (1 Kbp)**. Sentitivity-specificity operating characteristics curves for RAIphy determined with 1 Kbp fragments using the dataset obtained from the RefSeq database. The accuracy values for TACOA and PhyloPythia are also illustrated for the same test data.

**Figure 9:**
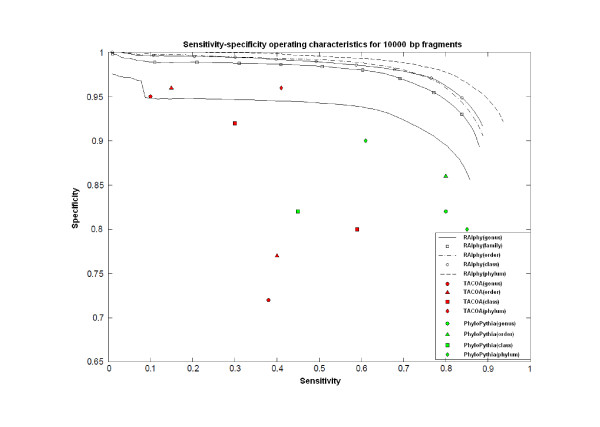
**Sensitivity-specificity operating characteristics (10 Kbp)**. Sentitivity-specificity operating characteristics curves for RAIphy determined with 10 Kbp fragments using the dataset obtained from RefSeq database. The accuracy values for TACOA and PhyloPythia are also illustrated for the same test data.

Figures 7, 8, and 9 show the specificity-sensitivity performance obtained from a cross-validation test on the dataset for 800 bp, 1 kbp, and 10 Kbp fragments (The specificity results for each taxon in 800-50 kbp fragment range are illustrated in Additional File 5). Four thousand random fragments were sampled from each test species. The optimized sensitivity and specificity values for TACOA and PhyloPythia were also shown for the same datasets. RAIphy significantly outperformed both algorithms for the given range of fragments and clade-levels. An advantage of RAIphy, as demonstrated by the sensitivity-specificity performance curves, is that even when samples with low confidence scores are included in the classification, we retain high specificity; and the number of unknown samples decreases and sensitivity values increase, whereas the specificity drop is only around 10-25% for 800 bp and 1 Kbp fragments and around 10-15% for 10 Kbp fragments.

### Performance on Real-Life Metagenomic Data

**Figure 10:**
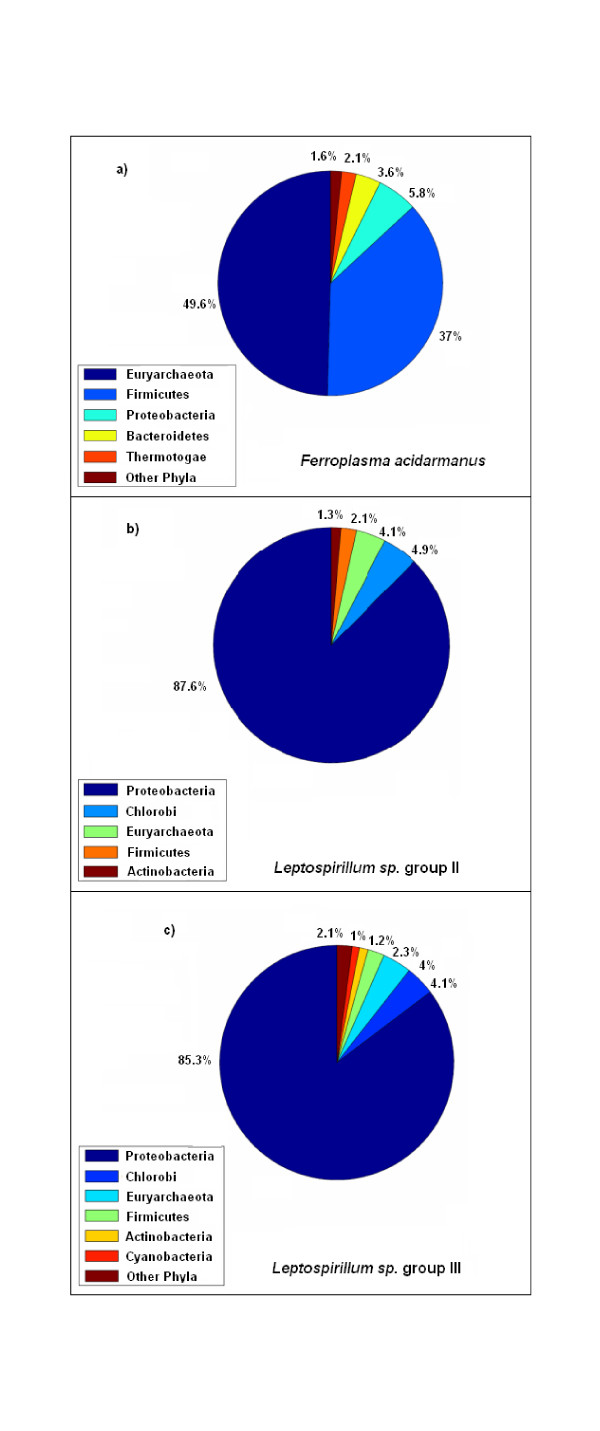
**Analysis of AMD dataset, Ferroplasma acidarmanus**. Phylum-level classification of the genome fragments belonging to *Ferroplasma acidarmanus *(a), *Leptospirillum *sp. group II (b), and *Leptospirillum *sp. group III (c) according to the sequence alignments with the reads and draft the genome. For *Ferroplasma acidarmanus*, 49.6% of the reads are correctly classified as Euryarchaeota. For *Leptospirillum sp*. groups II and III, 87.6% and 85.3% of the reads are classified as Protobacteria respectively.

The RAIphy system was also tested using a real-life dataset. Recognizing the control on real metagenome data is very limited and that true labels of assembled contigs and reads are not entirely known or the labeling is low quality, the experiment was performed on a subset of an Acid Mine Drainage (AMD) metagenome [33]. The AMD sample consisted of a low-diversity community that was dominated by three microbic populations: *Ferroplasma acidarmanus *and *Leptospirillum sp*. groups II and III. Since these organisms exist abundantly in the community, it has been possible to assemble draft genomes for these organisms. Therefore, we can accurately determine which fragment reads belong to these organisms with sequence alignments since fragments originating from the draft genomes align with few mismatches. This allowed us to observe the classification accuracy of our method for a subset of real metagenome data that could be accurately labeled. The phylum-level taxonomy assignments for each of the three genomes are shown in Figure 10. *Ferroplasma acidarmanus *belongs to Euryarchaeota phylum of Archaea; 49.6% of the fragments were correctly classified, as shown in Figure 10-a. This compares with 41.4% for Phymm, 48.6% for MEGAN, and 61% for PhymmBL, as shown in Table 1. The similarity scores used as MEGAN input were obtained from nucleotide BLAST with the RefSeq database used as the similarity search set.

**Table 1:** AMD dataset analysis comparison Ferroplasma acidarmanus

PHYLUM	Phymm	MEGAN	PhymmBL	RAIphy
Euryarchaeota	41.4%	48.6%	61%	49.6%

Firmicutes	41.9%	18.9%	28.8%	37%

Proteobacteria	8.6%	17.1%	4.9%	5.8%

Bacteroidetes	3.7%	2.2%	2.7%	3.6%

Thermotogae	1.8%	1.2%	<1%	2.1%

Other phyla	2.6%	12%	2.6%	1.9%

Phylum-level classification of the genome fragments belonging to *Ferroplasma acidarmanus *according to the sequence alignments with the reads and draft of the genome for the taxonomic classification programs Phymm, MEGAN, PhymmBL, and RAIphy. Correctly classified phylum is Euryarchaeota.

*Leptospirillum sp*. groups II and III are bacteria belonging to the Nitrospirae phylum, which does not exist in the NCBI RefSeq database and, consequently, in our database. The genus *Leptospirillum *was assigned as Deltaprotobacteria [34], which is a class of Protobacteria. Of the fragments putatively determined to be *Leptospirillum sp*. group II reads, 87.6% were assigned to the Protobacteria phylum. For Phymm the true positive percentage was 80.2%, for MEGAN it was 60.4%, while for PhymmBL it was 79.6%, as shown in Table 2. Finally for *Leptospirillum sp*. group III fragments, the true positive rate for RAIphy was 85.3%. This compares to 77.3% for Phymm, 62% for MEGAN, and 76.9% for PhymmBL, as shown in Table 3. This is a significant improvement in classification performance.

**Table 2:** AMD dataset analysis comparison Leptospirillum sp. group II

PHYLUM	Phymm	MEGAN	PhymmBL	RAIphy
Proteobacteria	80.2%	60.4%	79.6%	87.6%

Chlorobi	6%	2.5%	5.7%	4.9%

Firmicutes	2.3%	10.2%	2.7%	2.1%

Actinobacteria	<1%	1%	2%	1.3%

Other phyla	2.6%	12%	10%	4.1%

Phylum-level classification of the genome fragments belonging to *Leptospirillum sp*.group II according to the sequence alignments with the reads and draft of the genome for the taxonomic classification programs Phymm, MEGAN, PhymmBL, and RAIphy.

**Table 3:** AMD dataset analysis comparison Leptospirillum sp. group III

PHYLUM	Phymm	MEGAN	PhymmBL	RAIphy
Proteobacteria	77.3%	62%	76.9%	85.3%

Chlorobi	3.9%	1.7%	3.3%	4.1%

Euryarchaeota	8.4%	4.9%	7.7%	4%

Firmicutes	2.7%	6.8%	2.9%	2.3%

Actinobacteria	2%	12.7%	3.3%	1.2%

Cyanobacteria	1.1%	3.8%	1.3%	1%

Other phyla	4.6%	8.1%	4.6%	2.1%

Phylum-level classification of the genome fragments belonging to *Leptospirillum sp*.group III according to the sequence alignments with the reads and draft of the genome for the taxonomic classification programs Phymm, MEGAN, PhymmBL, and RAIphy.

## Conclusions

We proposed a metagenome binning method that exploits inherent features of genomic signatures with a novel measure called RAI and a novel classification metric. Our simulations used a large genomic fragment length range from 100 bp to 50 Kbp. This range covers the length of average metagenome assembly contigs and the length of sequencing reads with the current sequencing technology. The simulations resulted in classification accuracy ranging between 38-97% at the deepest clade-level (genus). Using RAI scores, the optimal performance was obtained using relatively longer oligonucleotides (7-mers) than methods using Euclidean distance and correlation-based scores utilizing shorter k-mer statistics. We attributed a part of the improvement in classification accuracy to being able to use longer oligonucleotide statistics, which include additional information on the DNA k-mer distribution. Moreover, with the availability of RAI profile updates using the predicted DNA sequences, we have defined an iterative classification method that improves the classification accuracy. We believe the improvement is due to the fact that genome signatures are pervasive, and genome models can be approximated without requiring the availability of complete genomes. Therefore, a small set of genome fragments was sufficient to update the initial genome models. In our case, a set of fragments forming 25 Kbp of nonoverlapping genomic sequence was sufficient to increase the classification accuracy in the next iteration.

In addition to the experiments performed on synthetic metagenomics data, we tested RAIphy with well-studied, real-life metagenome AMD sample reads. RAIphy outperformed the composition-based Phymm and nucleotide BLAST search-based MEGAN on the binning task. PhymmBL, which uses a composite method consisting of Phymm and BLAST, did better than RAIphy in one of the three tasks and worse in the other two. PhymmBL took substantially longer to complete the tasks than Phymm or RAIphy. The running time of RAIphy scales linearly with the average fragment length and the number of fragments in the metagenome sample. In our experiments, it took less than 4 hours to bin the AMD metagenome with the most comprehensive search models that contained all 1,146 genomic sequences of the (NCBI) RefSeq database on a standard desktop computer with a 2.19 GHz CPU. Processing of the same dataset with similarity-search-based binning programs, such as CARMA and MEGAN (run with blastn), and even with phylotyping pipelines AMPHORA and MLTreeMap, requires >24 hours. PhymmBL took around 464 hours to process the dataset. Using genus level RAI profiles, the current version of RAIphy can bin 1.5 Gbp of genomic sequences with 400 bp average read length in 24 hours. This amount of data is achievable with next generation, high-throughput sequencing; and RAIphy appears to satisfy a computational need for fast and accurate metagenome binning. RAIphy uses a moderate amount of memory (304 MB with species-level training loaded and 47 MB with genus-level training loaded) in its runtime.

We have implemented RAIphy as an open-source desktop application supported with a simple graphical user interface. While the default is for all the RAI profiles of the RefSeq database in the species and genus level to be used as database files, there is also an option to create custom databases if a set of training sequences are provided. Since the program performs with a satisfactory accuracy both for read-length and assembly-length DNA fragments, it can be utilized either as a preprocessing stage in a metagenomics pipeline to improve the assembly procedure or as the binning procedure for the assembled contigs.

We have observed that the accuracy falls to below 50% when sister taxa of the unknown fragments are not close relatives. This appears to be a universal problem that is also observed with other binning methods. For the metagenome samples of undiscovered microbes, it might be a safe strategy to sacrifice prediction resolution and bin the sequences to higher taxonomic units, such as phylum or class, or sacrifice specificity by selecting best hits and leaving suspicious assignments "unknown." RAIphy outputs assignments at all taxonomic levels as well as providing a thresholding option to select the best hits. Another universal problem, which RAIphy also suffers from, is the classification of horizontally transferred regions in procaryotes. Since recently transferred regions differ in composition, predictions of those regions result in false binning.

## Availability

The binary files and source code for RAIphy can be downloaded from http://bioinfo.unl.edu/raiphy.php.

## Authors' contributions

OUN came up with the RAI concept and developed and implemented the algorithms and wrote the initial draft of the manuscript. SW wrote the production version of the RAIphy program. SHH provided the biological insight and contributed to the final manuscript. KS conceived the study and collaborated in the development of the algorithm and in preparing the final manuscript. All authors read and approved the final manuscript.

## Supplementary Material

Additional File 1 **Empirical distributions of RAI scores**. Histograms of the Relative Abundance Index scores are shown for different levels of phylogenetic closeness. A RAI profile is built for a species, and RAI scores calculated using this profile for a relatively close relative and a distant relative are considered. A close relative is expected to have higher RAI scores, and a low score is expected for a distant relative. The empirical distributions calculated by RAI score histograms support this claim. Click here for file

Additional File 2 **Analytical result showing RAI scores measures compositional similarity**. With the assumption that elements from the same taxonomic units follow the same K-mer probability distribution, it is shown that RAI profiles of DNA fragments from the same taxon attain higher membership scores than fragments from other taxa. Click here for file

Additional File 3 **Classification performance of RAI similarity measure**. Figure 1 shows the comparison of Relative Abundance Index measure with other similarity measures for 100 bp-1000 bp fragment lengths. An oligomer length of 7 is used. Figure 2 shows the detection accuracy for varying oligomer lengths (dinucleotide to octanucleotide) using an RAI measure in the range of 100 bp-1000 bp fragment length. Click here for file

Additional File 4 **Binning performance in the Absence of Close Relatives**. Comparison of RAIphy, BLAST, and Phymm with incomplete training set for varying clade-levels is shown for 100 bp, 200 bp, and 800 bp genomic fragments. Click here for file

Additional File 5 **Performance of RAIphy for longer genome fragments**. Using metagenome fragments ranging from 800 bp to 50 Kbp, the specificity results were illustrated for each taxon. The results are supplied for clade-levels of genus, family, order, class, and phylum seperately. Click here for file
