# Supplementary material for: RAIphy: Phylogenetic classification of metagenomics samples using iterative refinement of relative abundance index profiles
Source: BMC Bioinformatics. 2011 Jan 31;12:41. doi: 10.1186/1471-2105-12-41 (PMC3038895; doi:10.1186/1471-2105-12-41)
Supplement: Additional File 1 — Empirical distributions of RAI scores. Histograms of the Relative Abundance Index scores are shown for different levels of phylogenetic closeness. A RAI profile is built for a species, and RAI scores calculated using this profile for a relatively close relative and a distant relative are considered. A close relative is expected to have higher RAI scores, and a low score is expected for a distant relative. The empirical distributions calculated by RAI score histograms support this claim. [file 1471-2105-12-41-S1.PDF]

Empirical distributions of RAI scores

Here, histograms of the Relative Abundance Index scores are shown for different levels of phylogenetic closeness. Two RAI profiles are built for different species and membership scores are calculated and compared using these profiles using genomic fragments from a species that is phylogenetically closer to one of the profile source. A close relative is expected to have higher membership scores and low score is expected for a distant relative. The histograms are derived over 10000 random samples of 400 bp DNA fragments.

We observed the membership scores with fragments from varying phylogenetical relatives. Figure 1 shows membership score distributions of DNA sequences with respect to relatively close profile sources. The fragments belong to another strain of a species for which one of the RAI profile was calculated and the second profile was derived from another species in the same genus. The score distributions are observed to be close. Figure 2 shows membership score distributions of DNA sequences from moderately distant profile sources. The fragments belong to another species of a genus for which one of the RAI profiles was calculated. The second profile was calculated from another genus in the same family. The score distributions are observed to differ moderately. Figure 3 shows membership score distributions of DNA sequences with respect to distant profile sources. The fragments belong to another species of a genus for which one of the RAI profiles was calculated. The second set of fragments are from another phylum. The score distributions are observed to differ significantly.

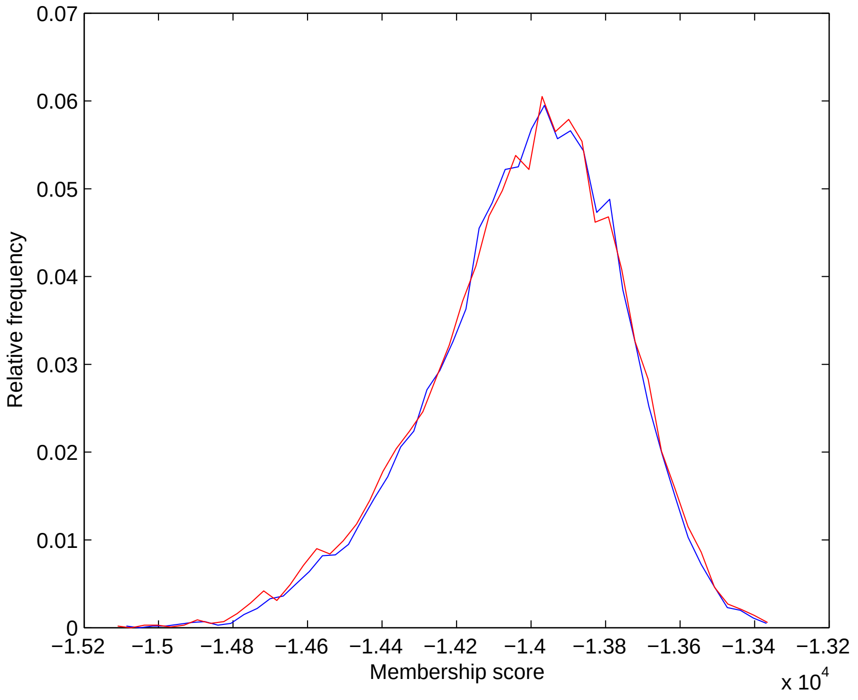

Figure 1: RAI profiles were derived from *Salmonella enterica subsp. enterica serovar Typhi Ty2* and *Salmonella typhimurium*. Random DNA fragments from *Salmonella enterica subsp. enterica serovar Typhi str. CT18* were used for score calculations. Blue histogram: Scores of fragments with respect to *enterica serovar Typhi Ty2* RAI profile. Red histogram: Scores of fragments with respect to *Salmonella typhimurium* RAI profile. Species from same genus show very close behaviors with the RAI profile in the same genus. Classification accuracy based on maximum score detection is % 53.25

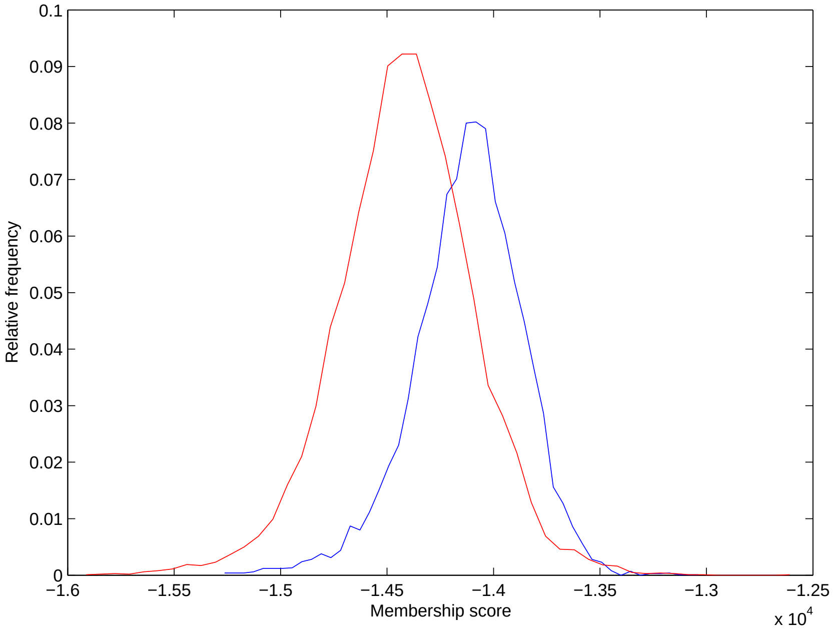

Figure 2: RAI profiles were derived from *Chloroflexus sp. Y-400-fl* and *Roseiflexus sp. RS-1*. Random DNA fragments from *Chloroflexus aggregans* were used for score calculations. Blue histogram: Scores of fragments with respect to *Chloroflexus sp. Y-400-fl* RAI profile. Red histogram: Scores of fragments with respect to *Roseiflexus sp. RS-1* RAI profile. Sequences from moderately distant relatives show a moderate difference in RAI (membership) scores. Classification accuracy based on maximum score detection is % 65.6.

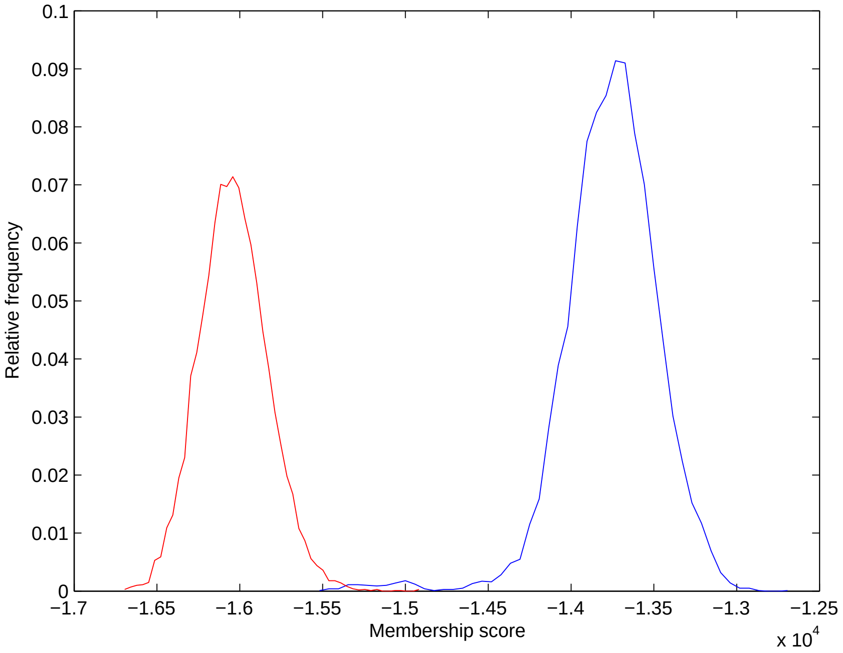

Figure 3: RAI profiles were derived from *Staphylococcus aureus* and *Pseudomonas aeruginosa*. Random DNA fragments from *Staphylococcus saprophyticus* were used for score calculations. Blue histogram: Scores of fragments with respect to *Staphylococcus* RAI profile. Red histogram: Scores of fragments with respect to *Pseudomonas* RAI profile. The first RAI profile is from Staphylococcus genus of Firmicutes, where the second one belongs to another phylum, Proteobacteria. Sequences from distant relatives show a significant difference in RAI scores. Classification accuracy based on maximum score detection is % 99.89
