# Supplementary material for: RAIphy: Phylogenetic classification of metagenomics samples using iterative refinement of relative abundance index profiles
Source: BMC Bioinformatics. 2011 Jan 31;12:41. doi: 10.1186/1471-2105-12-41 (PMC3038895; doi:10.1186/1471-2105-12-41)
Supplement: Additional File 2 — Analytical result showing RAI scores measures compositional similarity. With the assumption that elements from the same taxonomic units follow the same K-mer probability distribution, it is shown that RAI profiles of DNA fragments from the same taxon attain higher membership scores than fragments from other taxa. [file 1471-2105-12-41-S2.PDF]

Analytical result showing RAI scores measures compositional similarity

Here, we show our claim that DNA fragments belonging to the same group with a RAI profile is expected to have higher scores calculated using this profile than RAI scores they get using RAI profiles derived from a different taxonomical group. This observation is fundamental to our similarity measure. To observe this situation, we assume that K-mer frequencies from a group follows the same probability distribution for the DNA sequences in this group and different groups follow other probability distributions.

If the group α follows the K-mer probability distribution P_α, then the expected RAI score for the fragments of this group for the RAI profile of the same group turns out to be

$$E_F[rai^{G_\alpha}] = \sum_{\mathbf{x}} P_\alpha(x_1, x_2, \ldots, x_k) rai^{G_\alpha}(x_1, x_2, \ldots, x_k), \tag{1}$$

recalling that the RAI profile $rai^G(x_1, x_2, \ldots, x_k)$ is

$$rai(x_1, x_2, \ldots, x_k) = \sum_{i=0}^{k-2} rai_i(x_1, x_2, \ldots, x_k). \tag{2}$$

Therefore, the RAI score is

$$E_F[rai^{G_\alpha}] = \sum_{\mathbf{x}} P_\alpha(x_1, x_2, \ldots, x_k) \sum_{i=0}^{k-2} rai_i(x_1, x_2, \ldots, x_k), \tag{3}$$

Plugging in the RAI profile definition for the α fragment,

$$\begin{aligned}
E_{F_\alpha}[rai^{G_\alpha}] &= \sum_{i=0}^{k-2} \sum_{\mathbf{x}} P_\alpha(x_1, \ldots, x_k) \log_2 \frac{P_\alpha(x_1, \ldots x_k) P_\alpha(x_{k-1} \ldots x_{k-i})}{P_\alpha(x_k, \ldots x_{k-i}) P_\alpha(x_1, \ldots x_{k-1})} \\
&= \sum_{i=0}^{k-2} \{ \sum_{\mathbf{x}} P_\alpha(x_1, x_2, \ldots, x_k) \log_2 P_\alpha(x_1, x_2, \ldots x_k) \qquad (4) \\
&+ \sum_{\mathbf{x}} P_\alpha(x_1, x_2, \ldots, x_k) \log_2 P_\alpha(x_{k-1} \ldots x_{k-i}) \\
&- \sum_{\mathbf{x}} P_\alpha(x_1, x_2, \ldots, x_k) \log_2 P_\alpha(x_k, x_{k-1}, \ldots x_{k-i}) \\
&- \sum_{\mathbf{x}} P_\alpha(x_1, x_2, \ldots, x_k) \log_2 P_\alpha(x_1, x_2, \ldots x_{k-1}) \}.
\end{aligned}$$

We can simplify the equation (4) using the entropy definition $H(p) = -\sum P \log P$ and the marginalization property that (Sayood 2000) $\sum_{x,y} P(x, y) \log P(y) = \sum_y \log P(y) \sum_x P(x, y) = \sum_y P(y) \log P(y)$. Then

$$
\begin{aligned}
E_{F_\alpha}[rai^{G_\alpha}] &= \sum_{i=0}^{k-2}\{\sum_{\mathbf{x}} P_\alpha(x_1,x_2,\ldots,x_k)\log_2 P_\alpha(x_1,x_2,\ldots x_k) && (5)\\
&+ \sum_{\mathbf{x}} P_\alpha(x_{k-1}\ldots x_{k-i})\log_2 P_\alpha(x_{k-1}\ldots x_{k-i})\\
&- \sum_{\mathbf{x}} P_\alpha(x_k,x_{k-1},\ldots x_{k-i})\log_2 P_\alpha(x_k,x_{k-1},\ldots x_{k-i})\\
&- \sum_{\mathbf{x}} P_\alpha(x_1,x_2,\ldots x_{k-1})\log_2 P_\alpha(x_1,x_2,\ldots x_{k-1})\}\\
&= -\sum_{i=0}^{k-2}(H_k(P_\alpha)-H_{k-1}(P_\alpha)+H_i(P_\alpha)-H_{i-1}(P_\alpha)) && (6)\\
& && (7)
\end{aligned}
$$

where $H_i(P_\alpha)$ stands for the ith order entropy. Summing up the telescopic summation we obtain

$$
\begin{aligned}
E_{F_\alpha}[rai^{G_\alpha}] &= -\sum_{i=0}^{k-2}(H_k(P_\alpha)-H_{k-1}(P_\alpha)+H_i(P_\alpha)-H_{i-1}(P_\alpha)) && (8)\\
&= -(k-1)H_k(P_\alpha)-(k-2)H_{k-1}(P_\alpha)-H_{k-1}(P_\alpha)+H_{k-2}(P_\alpha)\\
&= -(k-1)H_k(P_\alpha)-(k-2)H_{k-1}(P_\alpha)-h_{k-1}(P_\alpha).
\end{aligned}
$$

In the equation (8), $h_{k-1}(P_\alpha)$ stands for the conditional entropy where $(k-2)$ previous bases are given in a (k-1)-mer.

Following similar steps, the RAI scores of the same fragments obtained using a β profile is evaluated as

$$
\begin{aligned}
E_{F_\alpha}[rai^{G_\beta}] &= \sum_{i=0}^{k-2}\{\sum_{\mathbf{x}} P_\alpha(x_1,x_2,\ldots,x_k)\log_2 P_\beta(x_1,x_2,\ldots x_k) && (9)\\
&+ \sum_{\mathbf{x}} P_\alpha(x_{k-1}\ldots x_{k-i})\log_2 P_\beta(x_{k-1}\ldots x_{k-i})\\
&- \sum_{\mathbf{x}} P_\alpha(x_k,x_{k-1},\ldots x_{k-i})\log_2 P_\beta(x_k,x_{k-1},\ldots x_{k-i})\\
&- \sum_{\mathbf{x}} P_\alpha(x_1,x_2,\ldots x_{k-1})\log_2 P_\beta(x_1,x_2,\ldots x_{k-1})\}
\end{aligned}
$$

for the each entropy component in the equation (8), we can use the property that average self information of a distribution is smaller than the cross entropy, which directly follows from the non-negativity property of Kullback-Leibler divergence of two probability distributions (Sayood 2000):

$$
H(P_\alpha) = -\sum P_\alpha \log(P_\alpha)) \leq -\sum P_\alpha \log(P_\beta)) = H(P_\alpha,P_\beta) \qquad (10)
$$

(since $D_{KL}(P_\alpha \parallel P_\beta) = \sum P_\alpha \log(\frac{P_\alpha}{P_\beta}) \geq 0$)

Thus, each component of the equation (8) is greater than the corresponding components of the equation (9). We obtain

$$E_{F_\alpha}[rai^{G_\alpha}] \geq E_{F_\alpha}[rai^{G_\beta}] \quad (11)$$

■.

References:

Sayood,K. (2000) *Introduction to Data Compression*, 2nd edn, Morgan Kaufmann, San Francisco, CA.
