# Supplementary material for: RAIphy: Phylogenetic classification of metagenomics samples using iterative refinement of relative abundance index profiles
Source: BMC Bioinformatics. 2011 Jan 31;12:41. doi: 10.1186/1471-2105-12-41 (PMC3038895; doi:10.1186/1471-2105-12-41)
Supplement: Additional File 3 — Classification performance of RAI similarity measure. Figure 1 shows the comparison of Relative Abundance Index measure with other similarity measures for 100 bp-1000 bp fragment lengths. An oligomer length of 7 is used. Figure 2 shows the detection accuracy for varying oligomer lengths (dinucleotide to octanucleotide) using an RAI measure in the range of 100 bp-1000 bp fragment length. [file 1471-2105-12-41-S3.PDF]

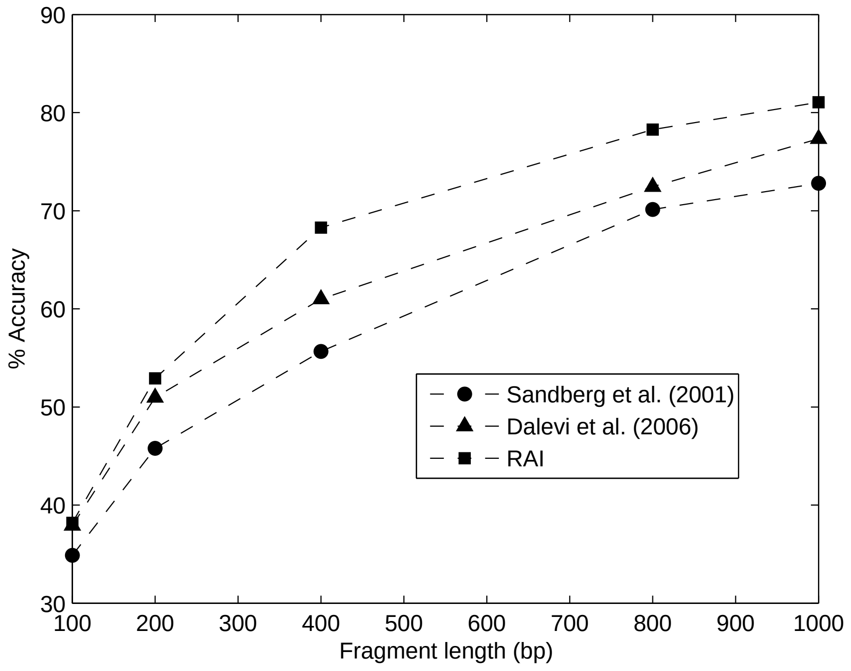


Figure 1: The comparison of Relative Abundance Index measure with other similarity measures for 100 bp-1000 bp fragment length. Oligomer length of 7 is used.

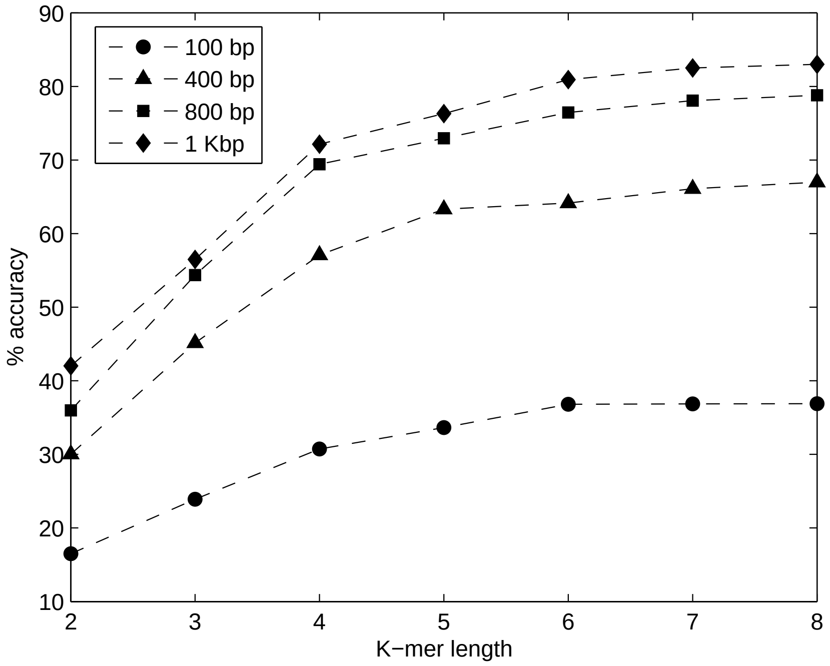


Figure 2: The detection accuracy for varying oligomer length using RAI measure in the range of 100 bp-1000 bp fragment length.
