# Supplementary material for: RAIphy: Phylogenetic classification of metagenomics samples using iterative refinement of relative abundance index profiles
Source: BMC Bioinformatics. 2011 Jan 31;12:41. doi: 10.1186/1471-2105-12-41 (PMC3038895; doi:10.1186/1471-2105-12-41)
Supplement: Additional File 4 — Binning performance in the Absence of Close Relatives. Comparison of RAIphy, BLAST, and Phymm with incomplete training set for varying clade-levels is shown for 100 bp, 200 bp, and 800 bp genomic fragments. [file 1471-2105-12-41-S4.PDF]

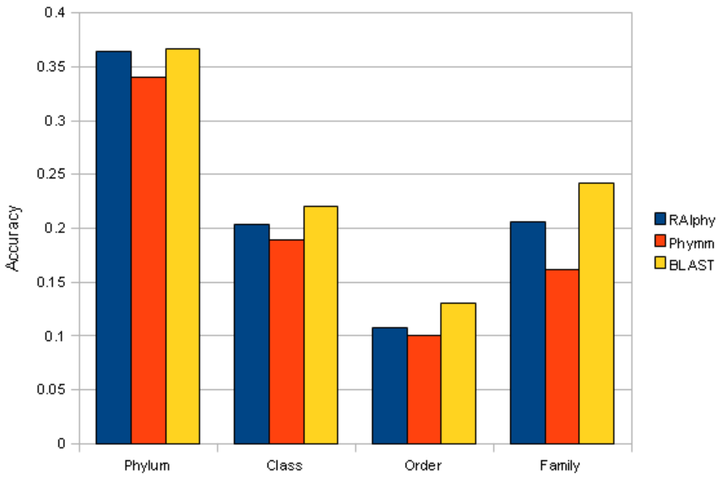

Figure 1: Comparison of RAIphy, BLAST and Phymm with incomplete training set for varying clade levels. Fragment length 100 bp.

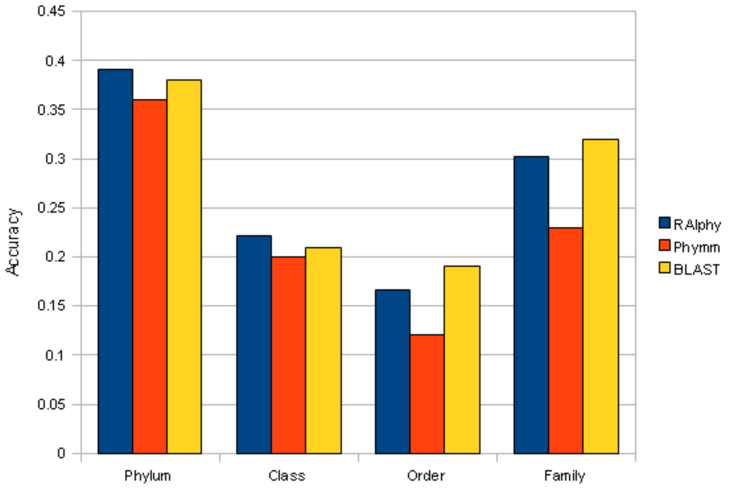

Figure 2: Comparison of RAIphy, BLAST and Phymm with incomplete training set for varying clade levels. Fragment length 200 bp.

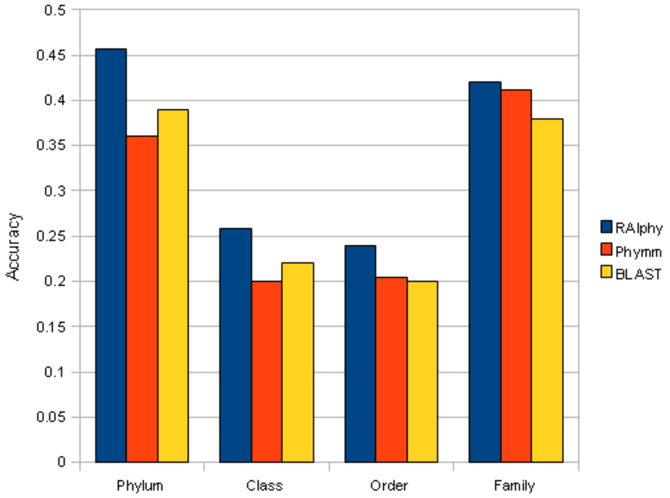

Figure 3: Comparison of RAIphy, BLAST and Phymm with incomplete training set for varying clade levels. Fragment length 800 bp.
