# Supplementary material for: RAIphy: Phylogenetic classification of metagenomics samples using iterative refinement of relative abundance index profiles
Source: BMC Bioinformatics. 2011 Jan 31;12:41. doi: 10.1186/1471-2105-12-41 (PMC3038895; doi:10.1186/1471-2105-12-41)
Supplement: Additional File 5 — Performance of RAIphy for longer genome fragments. Using metagenome fragments ranging from 800 bp to 50 Kbp, the specificity results were illustrated for each taxon. The results are supplied for clade-levels of genus, family, order, class, and phylum seperately. [file 1471-2105-12-41-S5.PDF]

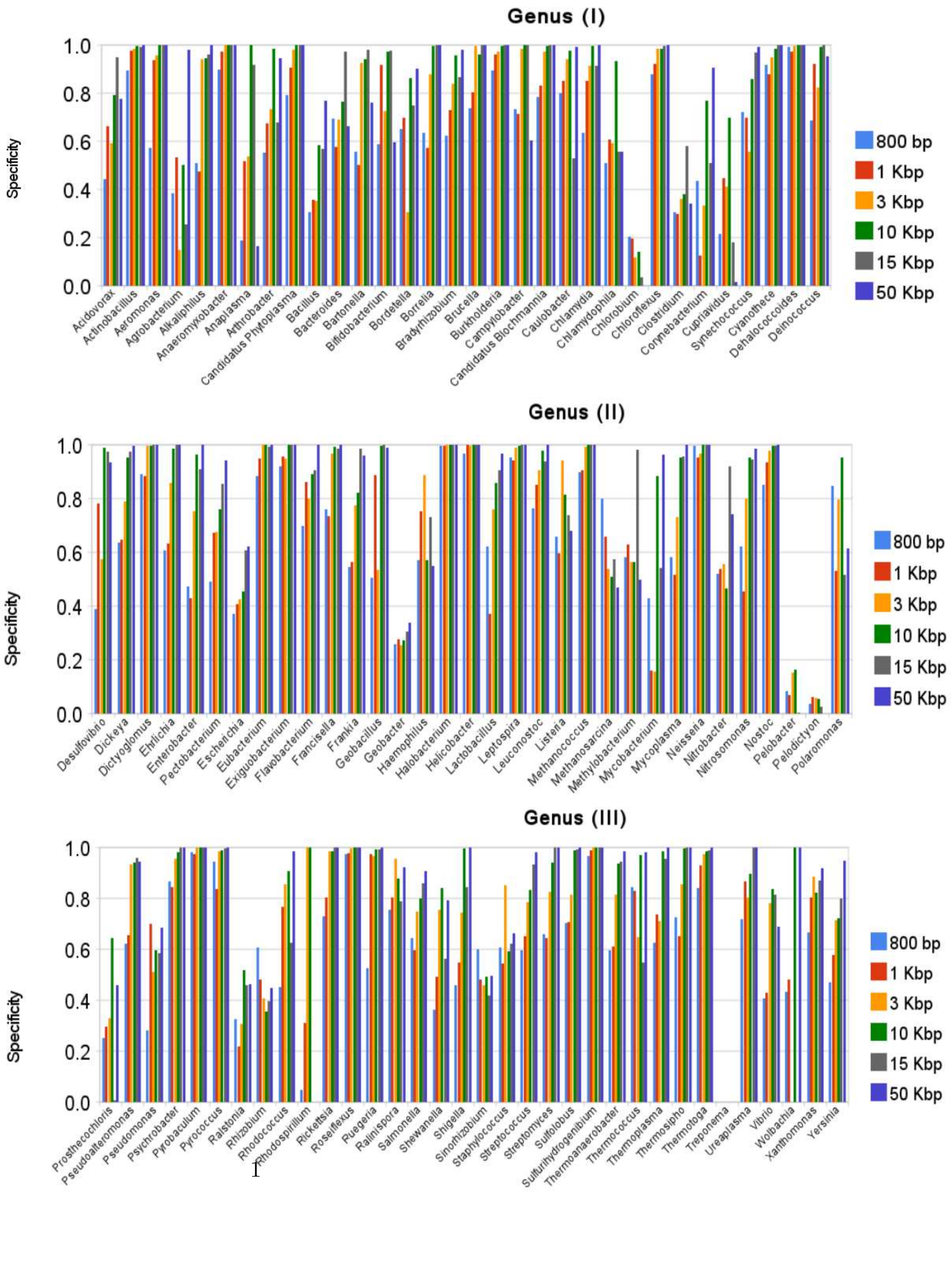

Figure 1: Specificity performance of RAIphy in genus level prediction for 99 genera obtained from RefSeq database. Fragment lengts of 800bp, 1Kbp, 3Kbp, 10Kbp, 15Kbp and 50Kbp are illustrated.

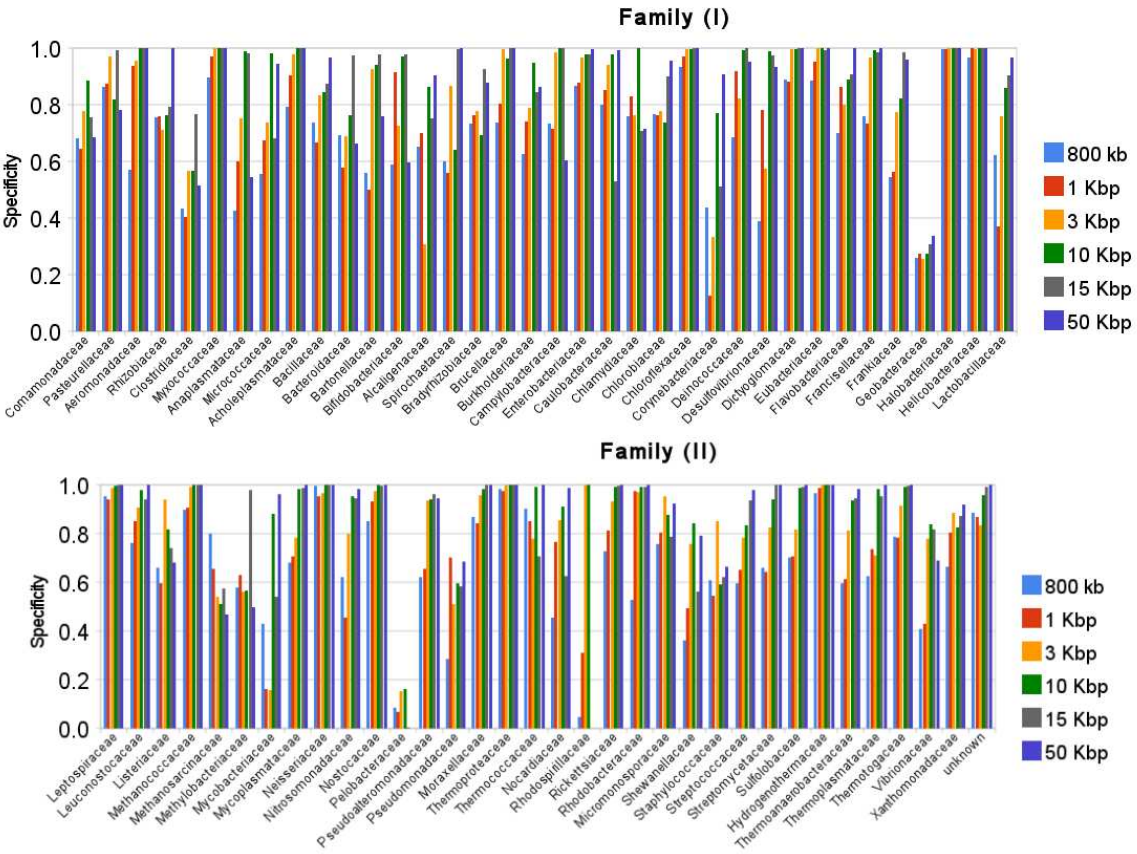

Figure 2: Specificity performance of RAIphy in family level prediction for 70 families obtained from RefSeq database. Fragment lengts of 800bp, 1Kbp, 3Kbp, 10Kbp, 15Kbp and 50Kbp are illustrated.

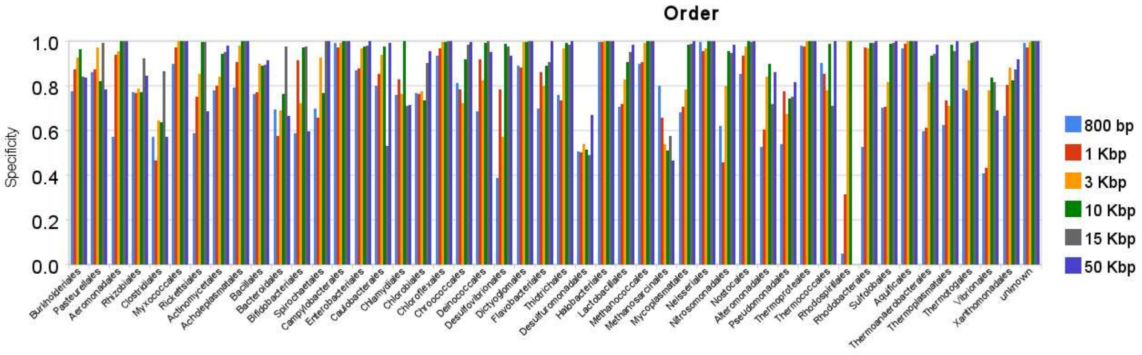

Figure 3: Specificity performance of RAIphy in order level prediction for 47 orders obtained from RefSeq database. Fragment lengts of 800bp, 1Kbp, 3Kbp, 10Kbp, 15Kbp and 50Kbp are illustrated.

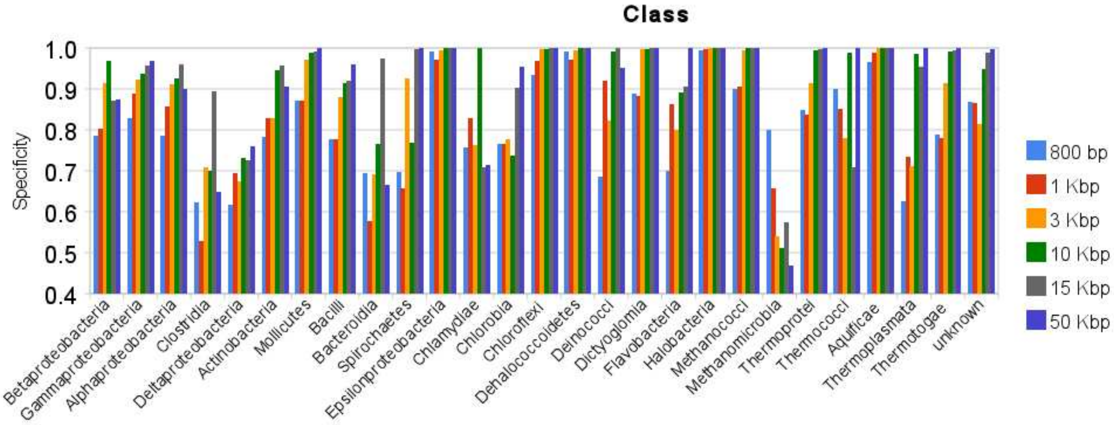

Figure 4: Specificity performance of RAIphy in class level prediction for 26 classes obtained from RefSeq database. Fragment lengts of 800bp, 1Kbp, 3Kbp, 10Kbp, 15Kbp and 50Kbp are illustrated.

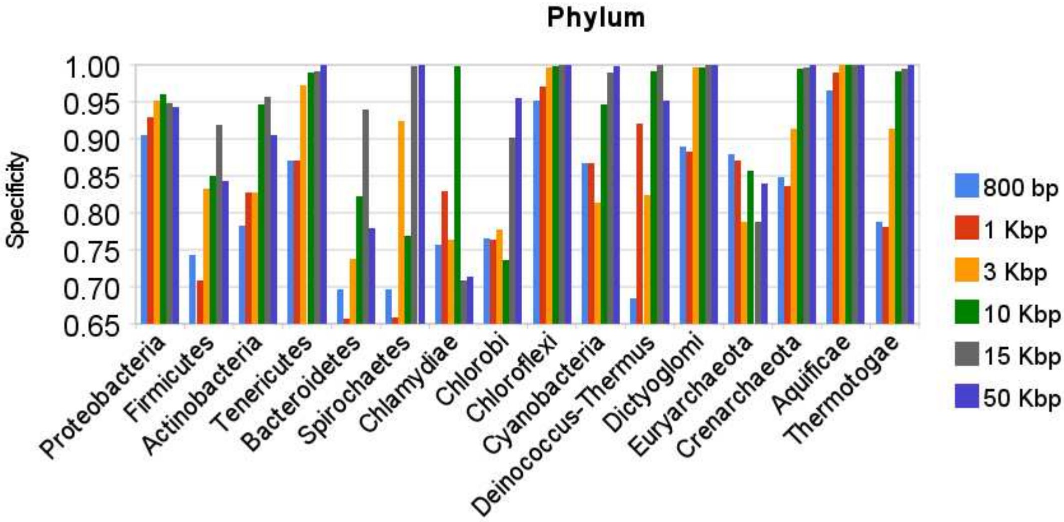

Figure 5: Specificity performance of RAIphy in phylum level prediction for 16 phyla obtained from RefSeq database. Fragment lengts of 800bp, 1Kbp, 3Kbp, 10Kbp, 15Kbp and 50Kbp are illustrated.
